# Balancing altruism, risk and ethics: Vietnamese students’ views on human challenge studies

**DOI:** 10.1371/journal.pone.0347941

**Published:** 2026-09-18

**Authors:** Chi Le Phuong, Chambrez Zita Zauchenberger, Vy Pham-Tram, Hong Huynh Thuy Phuong, Dong Thi Hoai Tam, Nguyen Minh Nguyet, Trung Dinh The, Thuy Nguyen Thi Van, Hang Nguyen Thi Thu, Thuan Hoang Van, Kien Vu Duy, Hung Vu Bao, Dung Do Van, Tuan Tran Diep, Bridget Wills, Jennifer Ilo Van Nuil, Evelyne Kestelyn

**Affiliations:** 1 Oxford University Clinical Research Unit, Ho Chi Minh City, Vietnam; 2 University of Medicine and Pharmacy at Ho Chi Minh City, Ho Chi Minh City, Vietnam; 3 Center for Global Health Practice and Impact, Georgetown University, Washington, District of Columbia, United State of America; 4 Centre for Tropical Medicine and Global Health, Nuffield Department of Medicine, University of Oxford, Oxford, United Kingdom; George Washington University School of Medicine and Health Sciences, UNITED STATES OF AMERICA

## Abstract

**Introduction:**

Human challenge studies (HCS) have been proposed to accelerate the development of health interventions and strengthen research capacity in low- and middle-income countries. However, implementation in settings such as Vietnam, where clinical care facilities and research capacity are limited and no specific regulatory framework for HCS exists, raises special ethical, social, and practical concerns. This study examined healthcare professional students’ perceptions of and attitudes toward HCS, focusing on ethical considerations and factors associated with willingness to participate.

**Methods:**

A mixed-methods study was conducted as part of a longitudinal project, named SEED (2020–2024) among students at the University of Medicine and Pharmacy, Ho Chi Minh City, Vietnam. Quantitative data was collected using a structured survey administered after engagement activities on HCS (n = 242). Qualitative data were obtained through focus group discussions (n = 54) and in-depth interviews (n = 14). Findings were integrated using an explanatory mixed-methods approach.

**Results:**

Participants reported moderate familiarity with HCS and expressed cautious support. Acceptability of HCS was lower than that of clinical trials or observational studies, reflecting concerns related to intentional infection. Motivations for participation included altruism, perceived social value, and potential national benefits, particularly for endemic diseases. Willingness to participate was positively associated with assurances of participant safety, access to rescue treatment, long-term compensation for research-related harm, transparent informed consent processes, and trust in research institutions and regulatory oversight. Concerns about potential health risks, psychological burden, time commitment, fairness of compensation, stigma, and family influence were significantly highlighted.

**Conclusion:**

Medical and public health students in Vietnam expressed conditional support for HCS, while balancing perceived benefits against ethical and practical concerns. Establishing clear governance mechanisms, risk mitigation strategies, and appropriate compensation frameworks will be critical for ensuring the ethical conduct and acceptability of HCS in similar settings.

## Introduction

Jamrozik et al (2021) define human infection challenge studies (HCS) as: *“experiments that involve infecting research participants with pathogens (or other micro-organisms) and primarily aim to (i) test (novel) vaccines and/or therapeutics, (ii) generate knowledge regarding the natural history of infectious diseases (and/or asymptomatic infection), and/or (iii) develop “models of infection”—i.e., reliable methods (to be used in studies with aims (i) and/or (ii)) of infecting human research participants with particular pathogens”* [1]. HCS have the potential to accelerate vaccine and therapeutic development and may lead to vital breakthroughs in our scientific understanding of poorly characterized diseases [1]. Over the past decade, there have been increasing calls to conduct HCS in low- and middle-income countries (LMICs) to produce relevant scientific advances on neglected diseases and social value for target populations in endemic settings [2–9]. In line with the potential risks of such studies for healthy volunteers, current HCS are conducted under critical research scrutiny and rigorous ethical oversight; however, some public health experts, ethicists, policymakers and investigators are concerned that conducting HCS in LMICs could present additional ethical challenges. For example, conducting complex HCS research in the context of limited clinical care facilities and technical capacity might increase the risk of health consequences for participants, especially those who may not have reliable access to affordable health services after the study ends [1].

In 2018, the Oxford University Clinical Research Unit (OUCRU) launched a series of engagement activities with a wide range of senior stakeholders from Vietnam and regional partners in South-East Asia (e.g., Ministry of Health, hospitals, ethics committees, academic institutions), with the aim of building a grounded understanding of HCS and exploring the scientific, ethical and practical issues to conducting HCS in Vietnam [8]. These experts emphasized that conducting HCS in Vietnam could advance the effort to develop vaccines and discover effective therapeutics to improve prevention and management of endemic diseases, such as dengue. However, while acknowledging the potential value of HCS, exposure of healthy humans to pathogens raises bioethical, social and practical concerns, especially since no legal/regulatory framework for such research currently exists in Vietnam [8]. To expand on these stakeholder discussions and involve the views of the wider health community, OUCRU collaborated with The University of Medicine and Pharmacy at Ho Chi Minh City (UMP) to conduct a longitudinal study named the SEED project, involving a cohort of Vietnamese medical and public health students from July 2020 to December 2024. The SEED project aimed to examine students’ perceptions of and attitudes toward clinical research, with a particular focus on ethical issues related to human challenge studies (HCS) in Vietnam [10]. This paper presents a component of the SEED project findings related to students’ perceptions and attitudes towards HCS, with a focus on ethical issues such as fair recruitment, fair compensation, informed consent and safety controls, and the factors that might influence their own decision-making around participating in such studies.

## Materials and methods

### Study design and setting

This work was conducted as part of the SEED project [10]. Within the SEED project, we enrolled 539 first- and third-year students from the faculties of General Medicine and Public Health between July 2020 to December 2024. Theses students were invited to a series of structured engagement activities continuing at intervals over 3 years in parallel with their academic studies at UMP and including seminars, science cafés, debates and role play events. These activities facilitated discussion of ethical issues in clinical research, such as informed consent, participant vulnerability, reimbursement, and compensation. The present analysis focuses on the SEED project’s phase during which we explored students’ attitudes toward HCS.

### Data collection

**Fig 1:**
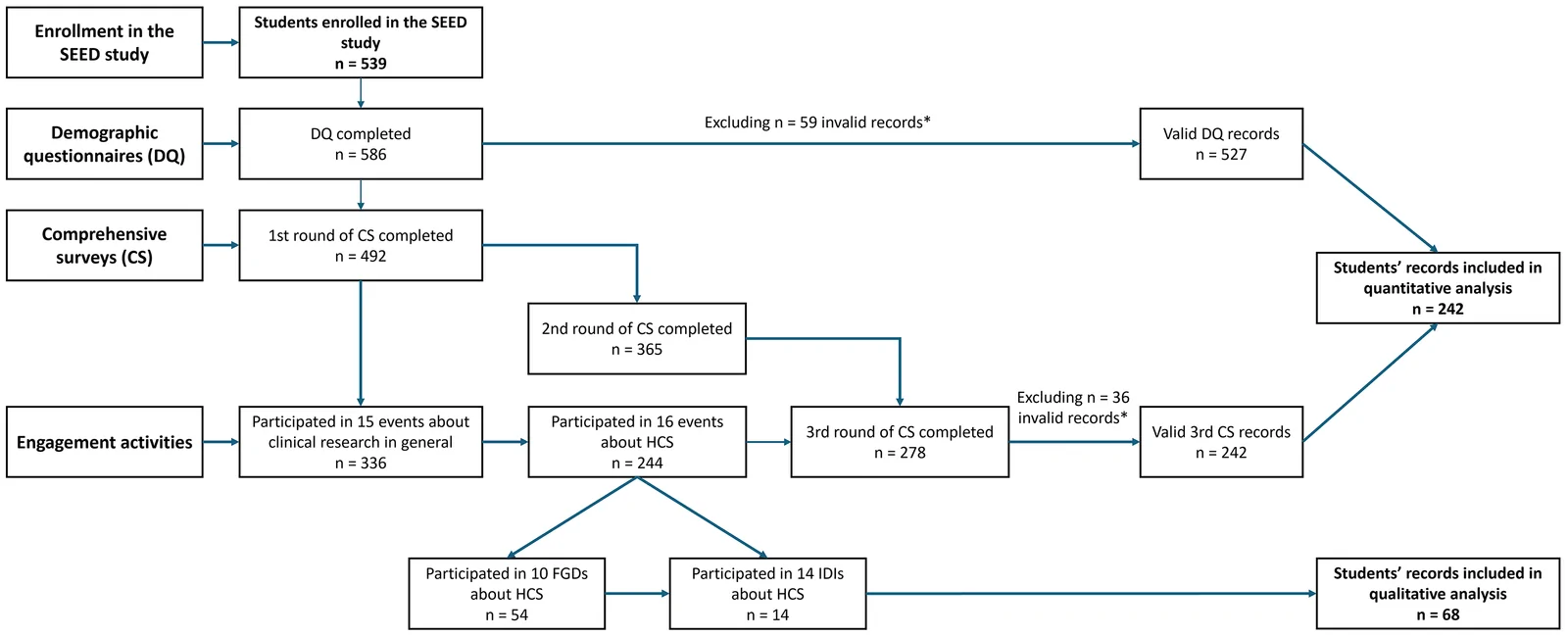
Flow chart of data collection. * Invalid records were excluded due to duplication and missing data.

We used a mixed-method study design administering a comprehensive survey (CS) alongside in-depth interviews (IDIs) and focus group discussions (FGDs) to collect quantitative and qualitative data on students’ opinions and attitudes towards HCS (Fig 1).

The CS (S1 Appendix) was administered at three timepoints during the SEED project, aiming to assess how students’ understanding of and attitudes toward various types of clinical research (observational studies, clinical trials, and HCS) evolved over time [10]. Results from the first round of the CS were reported on other publication [11]. This paper reports findings from the third survey, which began after initial HCS engagement activities and continued past the end of the entire engagement series.

We conducted 10 FGDs with a total of 54 students to collect a broad range of student perspectives, followed by IDIs with 14 students to explore individual experiences and expand on emerging themes. Topic guides were informed by relevant literature, and the questionnaires were then refined based on discussions generated in the science cafés, science debates and role play events on various issues related to HCS (S2 Appendix). Participants were purposively selected among students attending these engagement activities, to ensure diversity by gender, faculty, academic year, and socioeconomic background. Written consent was obtained from all participants prior to data collection.

### Data analysis

*Quantitative analysis:* The CS data were analyzed using R Statistical Software [12]. ChatGPT [13] was used only to refine and optimize R code for quantitative data analysis. We did not use any generative AI tools to generate data, interpret results, or create tables and figures. No study data were provided to the generative AI tools. All analysis outputs, research findings, visualizations, and conclusions were generated by the authors and reflected our own interpretations. Missing data accounted for less than 3% of responses; therefore, only complete cases analysis was used. For Likert scale responses, we collapsed from five to three categories where necessary to improve interpretability and model stability (Figs 3, 4A and 5) [14]. For the “select-all-that-apply” question (Fig 5), results are reported as the percentage of total respondents selecting each option. Descriptive statistics are used to summarize response patterns.

We used ordinal logistic regression (cumulative mixed-effect model, accounting for within-participant variation) [15] with random effects, to compare responses across study types, accounting for within-participant variation and the ordinal nature of the data. We adjusted P-values for multiple comparisons using the Benjamini–Hochberg false discovery rate (FDR) procedure [16] and considered FDR-adjusted p-values <0.05 as statistically significant (pFDR). Chi-squared tests for comparisons between two groups were applied where appropriate [17].

An overall acceptability score for each study type was calculated using five Likert-scale items assessing perceived importance, safety, willingness to participate, informed consent procedures, and incentives. These items were selected based on reliability testing (Cronbach’s alpha) and expert review [18]. Scores were converted to percentage of maximum possible (POMP) values using the formula: POMP=((observed score – minimum possible)/(maximum possible – minimum possible))X100, and the data were compared across study types using Wilcoxon signed-rank tests [19] (S3 Appendix).

*Qualitative analysis:* Qualitative data were managed using NVivo 12 and analyzed thematically using an approach informed by Braun and Clarke (2006) [20]. Interview recordings were transcribed, interpreted and analyzed in Vietnamese, with codes and themes documented in English.

### Ethics approvals

The study was approved by the Oxford Tropical Research Ethics Committee in Oxford, UK (approval number: 540−20 – dated July 2020) and the Ethical Committee of University of Medicine and Pharmacy at Ho Chi Minh City, Vietnam (approval number: 351/HDDD-DHYD – dated May 26, 2020).

## Results

**Fig 2:**
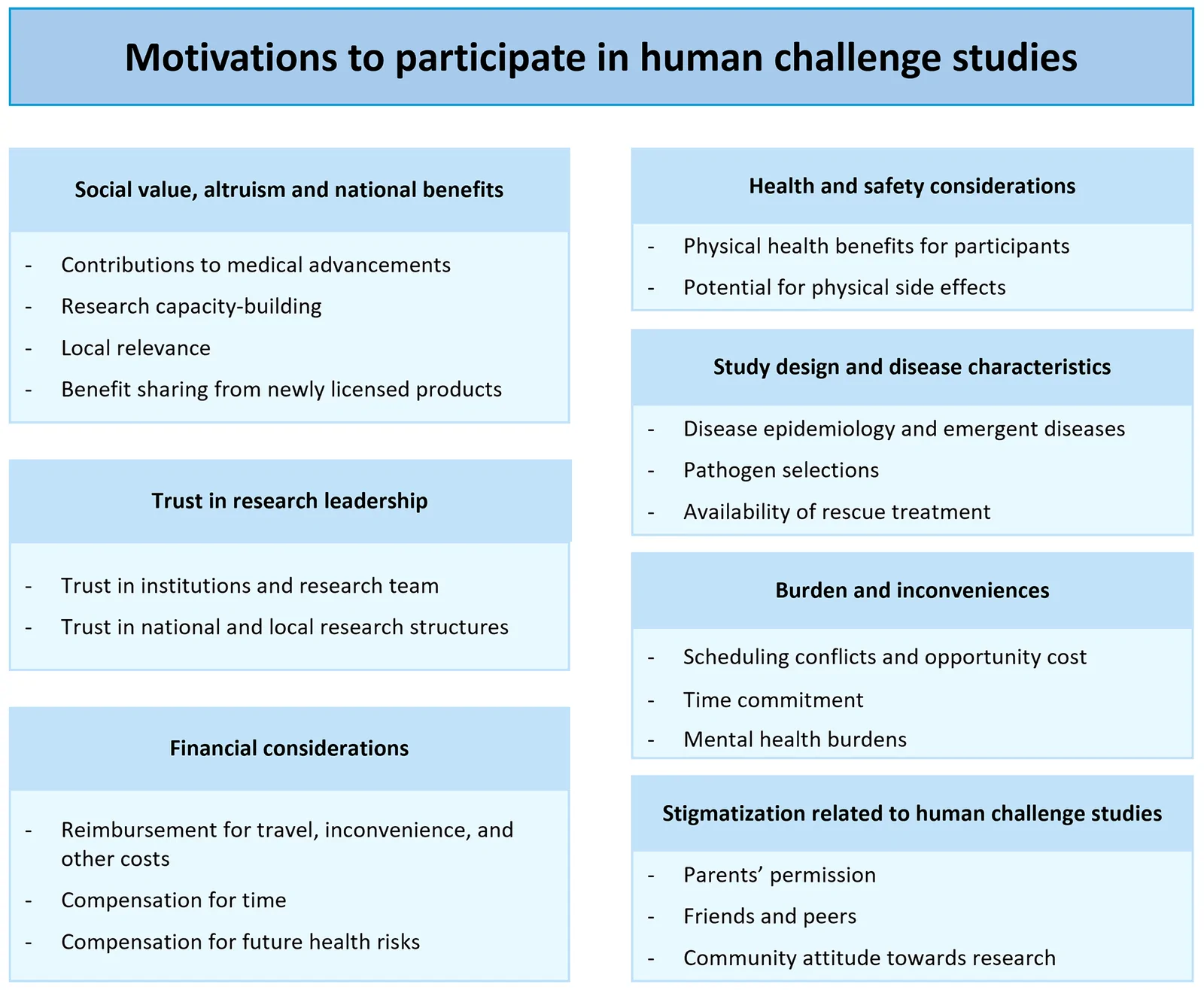
A framework for motivation factors of human challenge study participation.

We present first the demographic characteristics of students included in the analysis, followed by sections describing their overall knowledge of, and attitudes toward HCS in comparison with other research designs. Subsequent sections integrate survey and qualitative findings to outline the students’ motivations for participation in HCS and the key factors shaping their willingness to engage in such studies (Fig 2).

### Demographic characteristics of participants involved in this analysis

Results from 242 students are included from the CS after exclusion for duplication and missing data. Among this group, 68 students participated in either an IDI (n = 14) or FGD (n = 54). Demographic characteristics of students are presented in Table 1. The median age of the included students was 20 years, with balanced gender representation (50% male versus 50% female) and a predominance of general medicine students across faculties (64% general medicine versus 36% public health students). Most students (78%) self-reported an average socio-economic status, and the majority (93%) identified as Kinh ethnicity. The qualitative subsample broadly reflected the demographic composition of the larger survey cohort.

**Table 1:** Demographic characteristics of SEED cohort students included in the analysis.

	Quantitative data		Qualitative data	
	Comprehensive Survey		IDIs and FGDs	
**Total, n (%)**	**242**	**(100)**	**68**	**(100)**
**Age in years (median)**	20		20	
IQR	18-21		19-21	
**Faculty, n (%)**				
General Medicine	155	(64)	38	(56)
Public Health*	87	(36)	30	(44)
Preventative Medicine Department	46	(19)	10	(15)
Public Health Department	23	(10)	12	(17)
Nutrition Department	18	(7)	8	(12)
**Sex, n (%)**				
Female	122	(50)	35	(51)
Male	120	(50)	33	(49)
**Academic year at enrolment, n (%)**				
First year	116	(48)	32	(47)
Third year	126	(52)	36	(53)
**Ethnicity** ^**1**^ **, n (%)**				
Kinh	223	(93)	62	(91)
Hoa	9	(4)	2	(3)
Others	9	(4)	4	(6)
**Socio-economic status (self-reported)** ^**1**^**, n (%)**				
Poor	17	(7)	6	(9)
Average	184	(78)	47	(71)
Wealthy	2	(1)	1	(2)
Prefer not to answer	32	(14)	12	(18)

*: Public Health Faculty includes the departments of preventive medicine, public health and nutrition.

1: Missing data: In quantitative records: for ethnicity = 1; and for socio-economic status = 7; In qualitative records: for socio-economic status = 2.

### Students’ overall perceptions towards HCS

**Fig 3:**
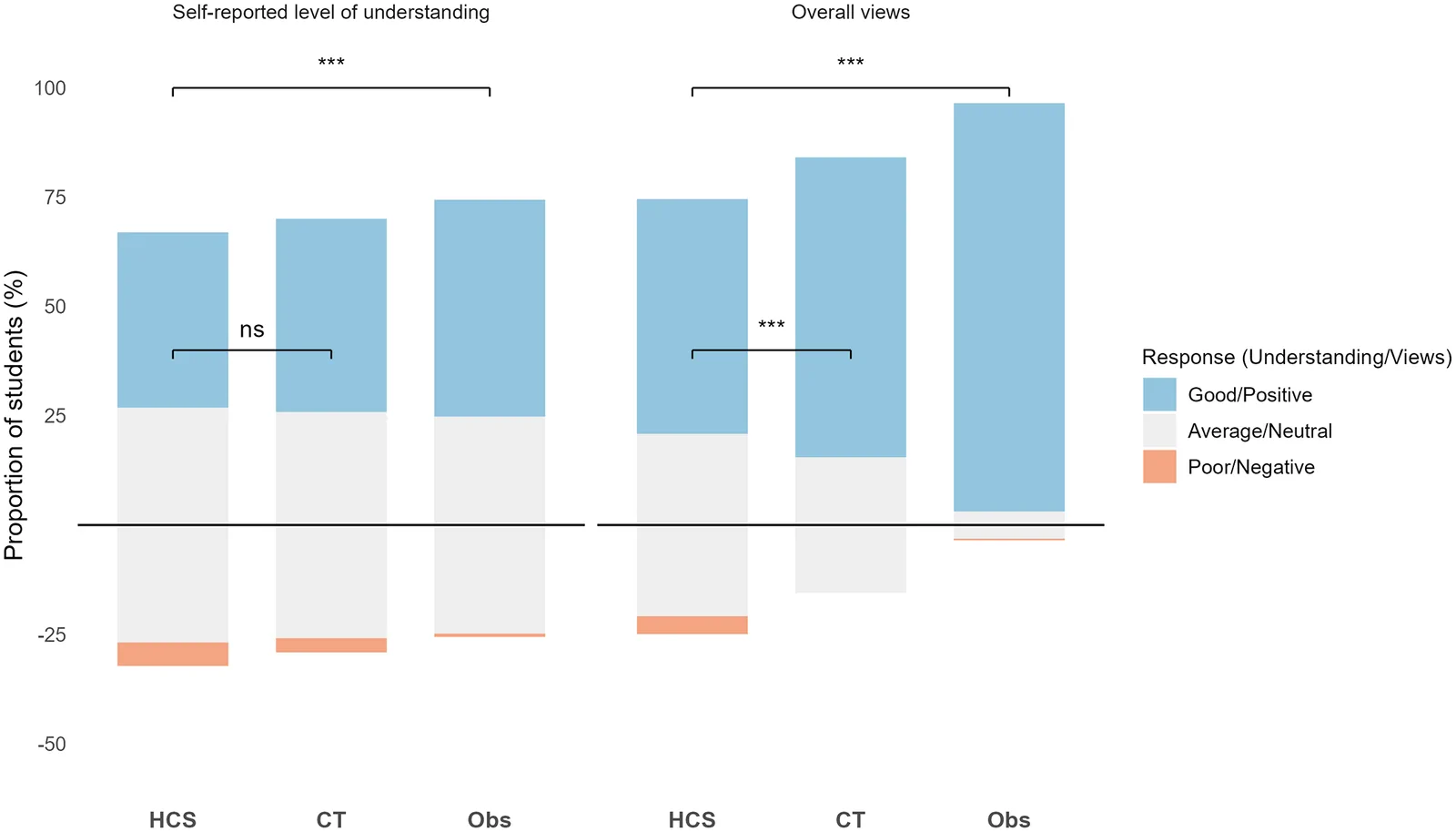
Students’ self-reported knowledge and overall views towards human challenge studies. This figure illustrates results from the comprehensive survey showing students’ self-reported understanding and overall views of human challenge studies (HCS), clinical trial (CT) and observational study (Obs). Two 5-point Likert scales assessing self-reported understanding (excellent, good, average, poor and very poor) and overall views (very positive, positive, neutral, negative and very negative) were collapsed to 3-point Likert scale (good, average and poor; and positive, neutral and negative) for analysis and visualization. The black line represents the midpoint of neutral responses. Response differences between study types (HCS vs CT; HCS vs Obs) were assessed using a cumulative mixed-effects model (predictor: study type, random intercept: participant) with false discovery rate (FDR) adjustment for multiple comparisons. *** pFDR < 0.001, ns: pFDR > 0.05.

The majority of students assessed their knowledge about HCS as being between average (54%) to good (40%). Overall, 54% of students reported positive views towards HCS, while 42% expressed neutral views (Fig 3). These findings suggest that while many students were familiar with the concept of HCS, strong endorsement was not universal, and a substantial proportion remained undecided or ambivalent.

**Fig 4:**
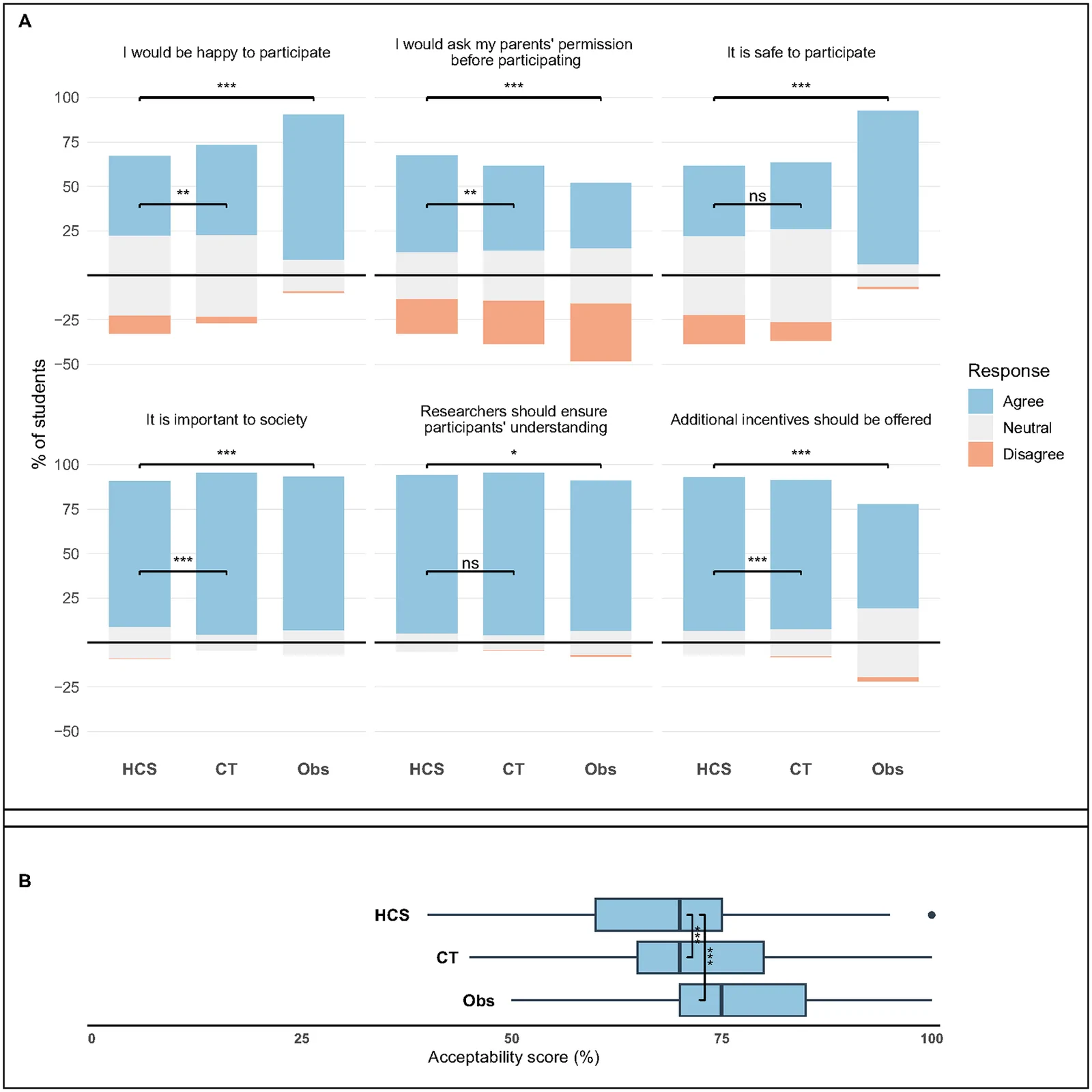
Students’ views on overall acceptability of human challenge studies. (A) This figure illustrates results from the comprehensive survey showing students’ perception and attitude toward human challenge studies (HCS), clinical trial (CT) and observational study (Obs). A 5-point Likert scale (strongly agree, agree, neutral, disagree and strongly disagree) was collapsed to 3-point Likert scale (agree, neutral and disagree) for analysis and visualization. The black line represents the midpoint of neutral responses. Response differences between study types (HCS vs CT; HCS vs Obs) were assessed using a cumulative mixed-effects model (predictor: study type, random intercept: participant) with false discovery rate adjustment for multiple comparisons. *: pFDR < 0.05; **: pFDR < 0.01, ***: p < 0.001, ns: pFDR > 0.05. (B) This boxplot represents the overall acceptability score of different trial types. Assessment of the acceptability score is presented in S2 Appendix. When comparing scores across two study type pairs, a Wilcoxon signed-rank test with continuity correction was used. ***: p < 0.001.

Comparative analysis of survey data demonstrated that HCS were viewed less favorably than more familiar forms of research (Fig 4A). Overall attitudes towards HCS (POMP score mean [SD]: 70.1% [12.3]) were significantly less strong compared with clinical trials (72.0% [11.0]; p < 0.001) and observational studies (77.1% [11.8]; p < 0.001) (Fig 4B). These differences indicate that, at a group level, HCS were perceived as more ethically or practically challenging than other research designs, even among students with training in health-related disciplines. No significant differences were observed in the acceptability of HCS among student group stratified by sex, faculty, academic year, ethnicity or social-economic status (Table 1 in S3 Appendix).

Qualitative findings revealed nuanced views regarding the perceived societal value, ethical acceptability, and comparative risks of HCS relative to other study designs. While many students expressed curiosity and interest in HCS as an innovative research approach, they also highlighted that limited prior exposure and uncertainty about risks made it more difficult to fully endorse HCS compared with conventional clinical trials or observational studies. Several students described their initial reactions as cautious or tentative, reflecting a process of weighing perceived social value against personal risk. This pattern of cautious openness aligns with the high proportion of neutral responses observed in the survey.

*“At first, since I did not have any knowledge [about HCS], I thought it [HCS] involved more risk than benefit… But after I learned about the medical purpose behind it [HCS] and the protection/ healthcare provided [to HCS participants], I would accept, I would agree to participate.” (FGD 56)*

### Factors influencing students’ motivation to participate in HCS

**Fig 5:**
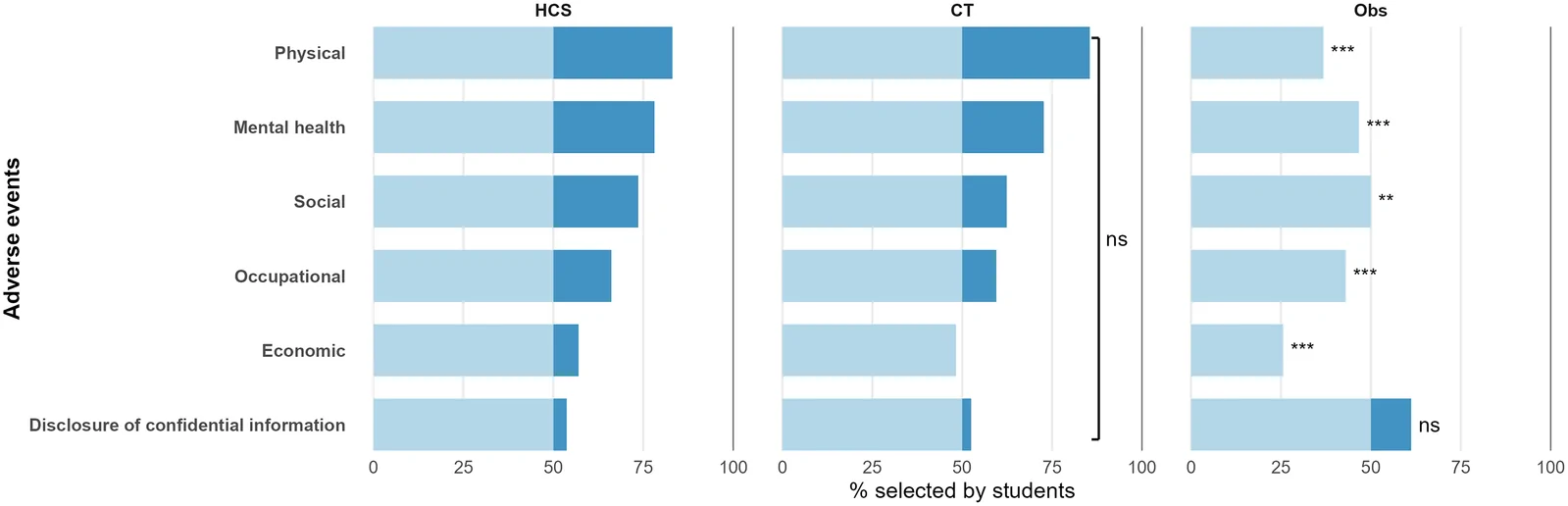
Students’ views on the risk and burden of human challenge studies. This figure illustrates results from the CS showing students’ views on the risks and burdens to participants in human challenge studies (HCS), clinical trials (CT) and observational studies (Obs). The percentage of students (N = 242) identifying each item is displayed, with values >50% highlighted for illustrative effect. Response differences between study types (HCS vs CT; HCS vs Obs) were assessed using Chi-squared tests with false discovery rate adjustment for multiple comparisons (*: pFDR < 0.05, **: pFDR < 0.01, ***: pFDR < 0.001, ns: pFDR > 0.05.), reported within the clinical trial and observational studies panels.

Because HCS have not been conducted in Vietnam previously, it was a novel concept for the majority of students who participated in this study. Drawing on both survey responses and qualitative data, this section outlines key considerations shaping potential participation, including social responsibility, altruism, perceived health related benefits, risks and burden, safety and health concerns, trust in research institutions and teams, financial considerations, and the influence of family, peers, and broader community attitudes (Fig 2). The CS results indicate that students’ willingness to participate in HCS was mixed, with 45% of students being happy to participate, while an equal proportion expressed a neutral view. (Fig 4A). Together, these findings underscore the complexity of participation decisions and the importance of contextual factors (Fig 5).

#### Social value, altruism, and national benefits.

Across both survey and qualitative data, the perceived social value of HCS emerged as a central motivation. In the survey, the majority of students (82%) agreed that conducting HCS was important for society (Fig 4A). In the IDIs and FGDs, students framed participation in HCS as a contribution to medical advancement and collective goods, with potential benefits extending to family members, communities, and future generations. Participation was often described as an act of kindness, social responsibility, or moral contribution, nessitating exceptional commitment, compared to more routine forms of research participation. They described HCS participants as individuals with strong scientific understanding or altruistic drives, sometimes likening them to scientists or societal role models rather than ‘ordinary people’.

*“If only thinking about the benefits [of the studies] for the community, I associate it [the participants] with scientists or people with intensive knowledge, who want to contribute to the world. I think only exceptional people will agree to participate for the sake of the community. As for normal people, they... of course they put their own personal interests first.” (FGD 56)*

This framing suggests that beyond a mere research activity, students viewed HCS participation as a symbolic act reflecting broader values of social contribution and professional identity, particularly within the context of health-related practices. Students identified several ways in which HCS could generate societal benefit in Vietnam. First, they emphasized the potential of HCS to generate scientific knowledge quickly and efficiently, including early identification of symptoms from the inoculation time (signs and symptoms that were sometimes missing in observational studies) and improved understanding of immune responses and pathogen–host interactions. Many students contrasted this perceived efficiency with the longer timelines associated with traditional vaccine or drug development pathways.

Second, students highlighted the importance of local relevance in developing medical interventions. They believed that testing vaccines or drug candidates within local populations would be better suited to local epidemiological, environmental, and population characteristics. This was seen as particularly important for diseases that are endemic or pose a significant burden in Vietnam. Additionally, students noted that people might be more willing to participate if the research rationale and outcomes were directly relevant to themselves or their relatives’ health.

*“When conducting a human challenge study among Vietnamese people, because the [epidemiological] conditions, environment, and the [characteristics of] people/our population are likely similar. If it [an intervention] is effective for this group of people [participants], it might also be effective for others.” (FGD510)*

Finally, students emphasized that another motivation to participate in HCS would be the belief that local communities would gain benefits from the implementation of HCS in Vietnam, such as access to newly licensed vaccines and drugs. Even if the studies yielded unexpected results or were unsuccessful, insights learned from their rollout could provide direction for future studies based in Vietnam and improve research capacity. The knowledge sharing and collaborative partnerships through HCS implementation would enhance autonomy in vaccine and drug production.

*“When doing research, we may identify mistakes, strengths or breakthroughs [from the current study] that could be applied to future projects.” (FGD 511)*

#### Health and safety considerations.

In addition to social value, students discussed health-related considerations as potential motivations for participation. Some students perceived that participation in HCS could, under certain conditions, involve lower health risks than acquiring infection naturally. Those “controlled” attributes of HCS design encompassed careful participant screening, close medical monitoring, immediate access to healthcare, and structured follow-up during the study period. Students also viewed pre-enrolment health assessments as a benefit, providing insights into their own health status and potential disease risks. For some, the possibility of acquiring immunity or antibodies through controlled exposure or vaccination was seen as an additional advantage.

*“Before joining the study, I should receive a comprehensive health check-up. Only healthy people are selected to recruit for the study. I am examined, so I will know what my health status is. After being infected with a virus or bacteria... a successful result is that I am cured. And I get antibodies against the virus or bacteria.” (FGD 55)*

However, these perceived benefits were often accompanied by caution. Survey data showed that 39% of students agreed they felt safe participating in HCS, while 44% expressed neutral views, indicating uncertainty rather than outright rejection. These perceptions were similar to those reported for clinical trials (pFDR = 0.42). Most students (83%) identified the potential for physical side effects as a key burden (Fig 5). In the IDIs and FGDs, students emphasized that they would consider participation if side effects were anticipated to be mild or moderate, short-lived, and unlikely to cause long-term harm. Conversely, they would decline participation if infection could lead to organ damage, disability, or death.

*“I would not participate in [studies about] diseases whose pathogens have a high ability to spread quickly and weaken the immune system. For example, it [the disease] has a similar mechanism to HIV – like immunodeficiency diseases, that suppress the immune system and make me susceptible to other diseases. Or diseases that are highly infectious, that have not been controlled yet.” (FGD 58)*

Anaphylaxis was frequently mentioned, reflecting concerns about unpredictable immune responses even among healthy individuals.

*“As I understand, in HCS, they [participants] will be exposed to a pathogen. So, they might be slowly affected by the pathogen. So, it might impact their health. Or sometimes if they have already had health problems, it [the pathogen] could interact or something similar, so it can cause combined effects on health.” (IDI 31 – Public health, third year, female student)*

#### Study design and disease characteristics.

While some students believed that attenuated strains might reduce risks, they also recognized potential scientific limitations, including concerns about whether attenuated pathogens accurately reflect real world infection dynamics when evaluating vaccine efficacy. Students further described how disease epidemiology influenced motivation. Some indicated they would consider participating in HCS for diseases they might experience naturally in the near future, whereas others prioritized disease severity, particularly mortality.

*“During the period of a lethal pandemic, the mortality rate is high, and people need to quickly find medication for treatment or prevention. I would participate [in HCS] even though I know it is high risk. But in cases where it [the disease/epidemic] is not dangerous, I might not participate.” (FGD 55)*

*“I would consider whether the disease has just emerged, or it has been around for a long time and now a vaccine [needs to be] found. For example, Mpox has just emerged recently, so if there is a trial to test its new vaccine on humans, I will not participate. But if it is a vaccine trial on dengue, I will definitely participate. Because although this disease [dengue] has been around for a long time, we haven’t found a vaccine yet.” (FGD 59)*

Students’ experiences during the COVID-19 pandemic strongly influenced their perceptions of uncertainties surrounding HCS. Many expressed reluctances to participate in HCS involving emergent infectious diseases, due to concerns about long-term consequences and limited understanding of disease mechanisms.

*“I know that once I participate in the trial, it could have side effects; it could have long-term complications. But I don’t know what the odds are, whether it will happen to me or not, so I think I’ll still be worried at some point.” (FGD 510)*

In such cases, students preferred non-pharmacological public health measures over personal participation in HCS. However, some noted that willingness might increase as their scientific knowledge about emergent diseases improved.

Some students indicated that when considering the various types of investigational products used in HCS (e.g., pathogens, vaccines and/or drug candidates), they were less inclined to participate in HCS that included a vaccine candidate, because of the possible increased risk related to interactions between investigational agents. They worried that interactions between a pathogen and a vaccine candidate (containing antigens or genetical material from the pathogen) could generate risky mutations or variants, potentially leading to severe complications for participants.

*“The virus that I am infected with first, and then the vaccine [against the virus] somehow could create a mutation. The new variant (viral mutation) could make the disease flare up and it will become more difficult to control.” (FGD 57)*

The availability of rescue treatment was another decisive factor. Students expressed greater willingness to participate when clear rescue or supportive treatment strategies were in place.

*“Even though there are some diseases for which specific therapeutic treatment is not yet available, still people [the investigators] can manage them [the symptoms/consequences]. I will prioritize if they [the investigators] have a rescue treatment regimen. And they have something like [a special] treatment regime if the patients get worse.” (FGD 58)*

Concerns about transmitting infection to others, particularly family members, contributed to hesitation. Although students recognized that strict infection control measures could minimize environmental risk, even a small possibility of onward transmission was viewed as ethically troubling.

#### Burden and inconveniences preventing HCS participation.

In the survey, more than half of students had concerns about HCS adverse effects on participants’ mental health (78%), occupation (66%), economic status (57%) and social life (74%). However, these perceived burdens were not specific to HCS, with no significant differences with clinical trials (pFDR = 0.744 for mental health effects; pFDR = 0.718 for occupation effects; pFDR = 0.565 for economic effects; and pFDR = 0.565 for social effects) suggesting broader anxieties about trial participation (Fig 5).

Time commitment, particularly compulsory quarantine duration, was a major barrier. Students preferred studies that aligned with their academic schedules or were conducted during holiday breaks.

*“For those who are studying, like me, or those are working, it is difficult to leave their jobs with a steady income and future [prospects] and then spend an extended time participating in a human challenge study and be isolated from society.” (IDI22 – General medicine, first year, male student)*

Mental health burdens were widely recognized. In the IDIs and FGDs, students indicated that the HCS participants might encounter stress and loneliness resulting from social disconnection, the lack of face-to-face contact, movement restrictions and the feelings of being physically separated from family and friends during the HCS quarantine period. Besides isolation, uncertainty about their own health status during participation could enhance mental health issues, especially for those with pre-existing mental health conditions.

#### Trust in institutions and research teams.

Trust emerged as a critical determinant of participation. Students evaluated trustworthiness based on the credibility of institutions, the competence and integrity of research teams, and the presence of robust regulatory oversight.

Reputation was central to institutional trust. Students expressed greater confidence in well-known institutions with strong research track records and established collaborations.

*“When I receive research [information] from a reputable unit, I believe that they have considered everything, from the professional scientific aspect to the social aspect.” (FGD 53)*

*“If the research unit is big, and they have support from foreign countries; they have collaborative ventures or something like that; their credibility is high, they have done many similar studies before, I will trust them more and I will find it easier to make decisions.” (FGD 510)*

Public visibility also mattered. Institutions recognized through media coverage or familiar to parents were perceived as more trustworthy.

*“If it’s an organization that is mentioned on television; (…) I watch them on the news; my parents know their name, and they are mentioned on the news, then it’s definitely a reputable organization. If it’s a strange organization, and when you search [its name] on the internet, there’s only one or two websites mentioning it. It’s like it’s not... On the surface, it shows that it’s not very reputable.” (FGD 55)*

Beyond institutional reputation, students considered the trustworthiness of the research team when deciding whether to participate in HCS. The students indicated that an investigator’s competence would be assessed by analyzing their educational qualifications, high-quality research experience, and credible performance history, especially in relation to HCS or the disease of interest for the study.

*“The expertise of the team that is conducting the research - The higher their educational level is and the more achievements they have, then I think the safer it [the study] will be.” (IDI 25 – Preventive medicine, first year, female student)*

The trustworthiness of the study team was also reflected in their research integrity, particularly through the investigators’ adherence to ethical principles, protecting participants’ confidentiality and respect for participants’ rights. For instance, students noted that how the investigators communicated with participants during the informed consent process would significantly influence their perceived trustworthiness. They would trust the researchers if the research information were communicated transparently. All research information, such as the study objectives, processes, benefits, risks, burdens and safety assurances, should be presented in visual formats (e.g., pictures and videos) alongside traditional written forms, using local language to ensure clarity and ease of understanding. The students stated that the manner in which the investigators communicated with participants should not mislead their risk-benefit analysis and decision-making around participation by concealing or downplaying potential risks. Furthermore, the students thought that the need for understanding tests to ensure that participants were able to give adequate informed consent was necessary. The survey data showed that majority of students (89%) agreed that researchers must check that participants really understand the details of the HCS research before they enroll them. These results were similar for clinical trials (91% agree) and observational studies (84% agree) and so represent the overall importance of this practice to the students (Fig 4A).

*“Explain everything to them [the participants]. And for example, at the beginning [of the study], if you [investigators] would not explain anything, but during their participation in the study, people suffered from risks that were not explained it at the beginning, I think they would be very frustrated. Moreover, it would affect the professionalism and reputation of the research team in the future.” (FGD 57)*

Additionally, the credibility of the research team could be determined through their commitment to past participants and accountability during the research process. Students indicated that positive feedback from participants in previous studies could indicate the research team’s trustworthiness.

#### Trust in national and local research structures.

Ethics and scientific committee oversight contributed substantially to trust. Students valued independent review as a safeguard for participant rights and safety. Collaboration with hospitals and government authorities further enhanced credibility, particularly given concerns about emergency care capacity.

*“It depends on the hospital sites that the research team collaborates with, and the level of collaboration between the research site and the local authorities. For example, if they [investigators] collaborate with the authorities, there would be no reason to be suspicious. But if the study is conducted independently, without any connection to a hospital or any government units, it would be untrustworthy.” (FGD 512)*

#### Financial considerations.

In the previous phase of the SEED project, we introduced students to the concepts of reimbursement, compensation, and incentive [11]. In this study, we further clarified their attitudes toward the impacts of these financial attributes on students’ motivation to participate in HCS.

Students agreed that participants should be reimbursed for expenses such as travel based on actual costs. However, expense-based reimbursement alone was not viewed as sufficient to motivate participation without broader assurances of safety and fairness.

Time committed to study procedures was perceived as a significant burden influencing student willingness to participate in HCS. Some students expressed concerned that the compensation for HCS participation might be inadequate, especially when considered lost income if part-time employment opportunities were compromised.

*“Because it seems like it [participating in HCS] takes my time from other things that I can do to make money.” (FGD 54)*

However, they emphasized that estimating fair compensation for time commitment to research was challenging. Although individualizing compensation based on participants’ personal requests could increase recruitment, differences in compensation would cause unfairness among participants.

*Student 1: For compensation, [I think] compensation should be individualized [for each participant]. (...) If there is something [which investigators suggest providing] that does not reach their expectation, they [the investigators] could review and see [negotiate] to meet the participants’ needs. Some people want it [compensation] to be high, but others might want it to be low. So, in general, from the company’s side, investigators could still balance the budget for compensation. I think it will be more attractive than giving a general deal.*

*Student 2: [I think] they [the investigators] shouldn’t individualize [compensation]. Because if it [compensation] is individualized, this [estimation measure] causes a kind of comparison between this person and that person. It means, in a study that they [the investigators] individualize [compensation]... if this person accepts higher risks, they will gain higher amounts of money. Then the study will not be unified. (FGD 51)*

Students strongly emphasized there should be adequate compensation, considered in the form of insurance, to cover the treatment costs for any future health consequences or impairments due to HCS.

*“Compensation is just an extra remuneration. But I feel that if someone agrees to participate in HCS, it means they are making a trade-off with their health. Therefore, they should receive well-deserved compensation; something equivalent, serving as a guarantee for their health.” (FGD 52)*

They indicated the need for a contingency compensation for potential loss of labor or ability due to health impairments resulting from HCS.

*“If I have any health problems [because of participating in the research], I want them [investigators] to fulfill my wishes about things that I could no longer do. (...) And, if I unfortunately die, I want them [the investigators] to take care of people around me. Those I want to take care of [i.e., family members] must be cared for and they [the research team] must ensure that these things are done for me. These things are the minimum level that I think it [compensation] must be enough to cover.” (FGD 51)*

Compensation for future health risk should be detailed and explained in the informed consent form and agreed upon before enrolment. However, even if this occurred, some students commented that they would not trust that they would be compensated in the future without receiving proof from former participants.

*“I think it is important to consider what the research team will do for the participants and how they will respond if impairment occurs. For instance, they should provide some specific examples so that participants can imagine to what extent the institution will support them, beyond just providing [a certain amount of money as] compensation. For example, they may commit in the consent form that they will follow-up the participant for a certain number of years, but [in reality] there might be no specific action showing what the institutions have done for previous participants. This means that if they just show things on paper, it is a bit too theoretical for patients, or participants to trust and participate [in such studies].” (FGD 51)*

Although students did not consider incentives as a primary motivator for themselves (because they did not view themselves as financially vulnerable), most (86%) agreed in principle that incentives should be offered (Fig 4A). Furthermore, they commented that if the research has many benefits to the community, their decision to participate would be influenced by their altruistic motives rather than financial incentives.

*“Financial benefits [Incentives] are like an add-on. It is added to the motives they [participants] put into the research.” (IDI 25 – Preventive medicine, first year, female student)*

#### Family, friends and community opinions about research and participation in HCS.

Family influence, particularly parents, played an important but not determinative role. While 54% of students in the survey indicated they would seek parental permission, many (19%) felt confident making independent decisions based on their medical training (Fig 4A). In the IDIs and FGDs, they indicated that their own educational background in healthcare gave them confidence to make independent decisions about their health. They could cross-check information about the study and study investigators from different sources, such as the media and internet, and/or ask advice from health professionals, especially those with whom they had strong relationships (e.g., family doctors, lecturers from university). Some students indicated they would be more likely to agree to participate in research if they knew that their friends or acquaintances, who had a similar level of medical understanding, were also participating.

*“I think I will look for [advice] from various sources. For example, first, acquaintances; second, I will ask people like doctors or someone like this. Or I mean acquaintances who have certain expertise. Then, people such as doctors, or people on the internet, or people having answers for what I want to ask. So, I want to search for information from different sources before making a decision.” (FGD 58)*

Students described balancing respect for parental concerns with their own assessment of risk. Strong parental opposition would likely lead to refusal to participate in HCS, whereas anticipated disagreement might result in non-disclosure.

Concerns about stigma and confidentiality influenced participation decisions. In the survey, 54% of students expressed concerns about the risk of their confidential information being disclosed through participation in HCS (Fig 5). These results were not significantly different from the findings for clinical trials and observational studies (pFDR = 0.852 for clinical trials and pFGR = 0.280 for observational studies), suggesting students view this as a risk or burden to research participation in general. In the FGDs and IDIs, when asked about how the views if the wider community might influence their willingness to participate in HCS, students emphasized that lay opinions would not be the primary factor, but that controversy surrounding community acceptance of HCS might cause hesitation. For example, there was particular concern about the potential for discrimination related to infection status, especially studies involving blood-borne or sexually transmitted diseases (e.g., HIV or Hepatitis B, C).

*“Considering things that might negatively affect me if I participate in the research, I think it would be society’s view towards me and my family. It’s my neighbors’ attitude. If I tell someone I have participated in testing this medicine or I have been infected with this virus, people will suddenly be wary of me. It feels... I think there’s a possibility that people will discriminate against me.” (FGD 59)*

## Discussion

This mixed-methods study provides one of the first empirical examinations of medical and public health students’ perceptions of HCS in Vietnam, a setting where HCS have not previously been conducted. Overall, the findings reveal a cautiously supportive but highly conditional acceptance of HCS among students, shaped by a complex interplay of altruistic motivations, risk perceptions, trust in institutions, and broader social and familial considerations. While students generally recognized the societal value of HCS and expressed moral willingness to contribute to scientific advancement and capacity building, this support was tempered by persistent concerns around safety, long-term health consequences, and the adequacy of ethical oversight and institutional accountability. Students had mixed views on the influence of disease-related factors (e.g., local prevalence of the disease, virulence of the investigational pathogen), research institutional trustworthiness, financial support, and peer and parents’ opinions on their willingness to participate in HCS. These findings align closely with reported motivations and decision-making considerations among HCS participants in various different settings like Thailand [21], Kenya [22,23], The Gambia [24] as well as the United States [25] and the United Kingdom [26].

### Altruism, moral identity, and conditional solidarity

Altruism emerged as a central motivator for hypothetical participation, with students framing participation as a moral contribution to society, scientific progress, and national development. This patriotic motivation was similarly reported by participants in studies conducted in Zambia [27] and Malawi [28]. These findings echo broader research showing that altruism and perceived societal benefit are leading motivations for research participation in LMIC contexts, alongside personal health benefits and access to care [29]. However, students’ altruism was conditional. They emphasized that the societal benefit of HCS must be directly relevant to the Vietnamese population, aligning with ethical consensus that local relevance and social value are core components of the justificatory framework for challenge studies in endemic or LMIC settings [1].

### Risk perception shaped by disease characteristics and lived experience

Consistent with findings from other HCS studies, perceived health risks were the most influential factor shaping students’ willingness to participate, particularly risks associated with pathogen virulence and investigational products [1,30]. Students identified a range of risk-mitigation strategies that would increase their comfort with participation, including the use of less virulent or attenuated strains, careful eligibility screening, availability of rescue treatments, and intensive clinical monitoring throughout the study. Such risk reduction strategies are well documented in the HCS literature [1,30–33].

However, students also implicitly acknowledged the trade-offs associated with these safeguards. Limited healthcare and research capacity for establishing safety measures and a controlled environment for HCS, which are common in LMICs, might entail the risk of delaying treatment, especially when therapeutic treatment for diseases is nonexistent or ineffective [1,33]. Further, the use of attenuated challenge strains or involvement of individuals previously infected with the HCS pathogen of interest might reduce the risk to participants (except for pathogens such as dengue) [23–25], but affect the ability to evaluate a vaccine candidate’s efficacy and thus influence the down-selection process [1]. Moreover, close monitoring and inpatient isolation could prevent serious complications or transmission to third parties but increase isolation burdens and restrict participants’ feeling of freedom to withdraw [33]. Investigators therefore face a delicate balance between generating scientifically robust data and minimizing participant risk and discomfort, reinforcing the need to consider HCS alongside complementary approaches to vaccine evaluation.

### Trust as a prerequisite for participation

Trust emerged as a foundational determinant of willingness to participate, operating at multiple levels: capable research teams, strong institutional governance, effective ethical oversight and national governance structures. Students’ endorsement of comprehensive consent and comprehension checks resonates with ethical analyses showing that informed consent in HCS must go beyond procedural formalities to ensure real understanding of risks, benefits, and study implications [29].

Students placed considerable weight on institutional reputation, collaborative partnerships, and public visibility as markers of credibility. These findings reflect broader evidence that trust in institutions and transparent governance are essential enablers of research participation, particularly in nuanced designs like HCS that involve intentional exposure [1,34]. WHO guidance underscores the need for robust regulatory and ethical oversight in HCS [31], yet Vietnam currently lacks HCS-specific regulatory and legal frameworks [8]. Although national guidelines for biomedical research ethics exist, they do not provide tailored guidance for reviewing HCS protocols. Given the evolving nature of HCS, continuous capacity strengthening for institutional review broad or ethics committee members, consultation with international experts, and early collaboration with policymakers are essential. Developing explicit criteria addressing strain selection, informed consent, endpoints, risk mitigation, and safety monitoring would support rigorous ethical review and guide investigators in aligning HCS protocols with national and international standards [31,35].

### (Fair) compensation, insurance, and moral boundaries

Students drew ethical distinctions between reimbursement, compensation for time, and compensation for potential future harm of HCS. While financial incentives were not primary motivators for this cohort, students strongly endorsed fair compensation frameworks that recognize risk, avoid undue inducement, and include contingency for future health consequences. Students expressed varied and sometimes inconsistent views on what constituted fair compensation, reflecting broader debates within HCS ethics [36]. Substantial variation in payment practices across HCS reflects the absence of a transparent and universally accepted framework for estimating compensation [37,38]. Recent guidelines recommend compensating HCS participants for time, inconvenience, and study burden, taking into account factors such as visit frequency, inpatient residence, and invasive procedures [31,37,38]. Frameworks proposed by Lynch et al. [38], which benchmark payments against comparable studies, local minimum wages, and additional burdens such as isolation or off-hours participation, offer practical guidance but require contextual adaptation.

Students were particularly concerned about compensation for potential future harms, emphasizing the importance of insurance or mechanisms to cover medical costs, lost income, suffering, or death—an ethical requirement in HCS [32]. Some participants supported upfront payment for future risk (the “payment for risk” model), a position that has garnered public support in other settings but remains controversial due to concerns about coercion and undue inducement [37,39].

In addition to reimbursement and compensation, offering incentives in HCS may be used to make research more attractive and encourage the participation of diverse population groups, but raise concerns about undue inducement due to attracting disproportionately individuals from worse-off economic backgrounds [38]. Incentives were generally viewed as having limited influence on students’ decisions to participate, although adequate compensation was seen as essential to feasibility. However, this finding must be interpreted cautiously, as students did not consider themselves financially vulnerable. Broader community engagement is therefore essential to understand how payment structures are perceived across diverse socioeconomic groups. Applying established clinical research payment frameworks, supplemented by clearer definitions of coercion, undue inducement, and ethically acceptable payment components in LMIC contexts, may help address these complexities [1,40–44]. Embedded social science research can further inform fair and transparent compensation strategies acceptable to participants, communities, and ethics reviewers alike and examine how these strategies align or are framed within other existing systems of research protection. Several authors highlight the importance of comprehensive and effective research protection frameworks in discussing the ethical challenges around participant reimbursements [45,46]. As Dresser (2001) [46] states: “*But payments are ethical only in the context of a comprehensive and effective system of research protections*.”

### Burden, mental health, and opportunity costs

Beyond physical risk, students identified significant psychosocial and practical burdens associated with HCS participation, especially concerns about isolation and mental health consequences. These concerns align with findings from broader clinical research participation studies, where opportunity costs and psychosocial burdens (e.g., time away from work or study) have been shown to meaningfully influence willingness to enroll [29]. Such insights suggest that HCS protocols must integrate robust psychosocial support and flexible scheduling to mitigate barriers to participation.

### Social context, family influence, and stigma

Despite their medical training, students remained embedded within familial and social networks that influenced decision-making. Many students indicated a preference not to disclose HCS participation to family or peers, primarily due to concerns about being perceived as infectious or posing a transmission risk. This mirrors findings from other HCS settings, where participants often conceal involvement to avoid social stigma [22,23,25]. While participants in a malaria HCS conducted in Thailand expressed concern about being associated with financial hardship [21], students in this study were more focused on infection-related stigma. Studies of research participation in LMIC contexts highlight how social endorsement or disapproval—particularly fear of discrimination for certain diseases—can meaningfully deter engagement, underscoring the importance of community engagement strategies to build social support for novel research designs [27]. Prior research conducted by OUCRU has highlighted the central role of public engagement in building trust and facilitating open dialogue around HCS in Vietnam [8]. Our findings reinforce the importance of embedding social science research and inclusive stakeholder engagement within HCS design and implementation to align studies with local ethical norms and foster and improve public acceptability and trust.

### Limitations

A limitation of this study is that students were provided only with a broad overview of HCS concepts rather than a detailed protocol, including specific information on study procedures, investigational disease pathogens and products, levels of risk, safety controls, and treatment. Limited study information might lead to discrepancies between our findings and the actual behaviors of potential participants. Additionally, our findings reflecting perspectives of the students, who were young, healthy and trained in medicine or public health, may not be generalizable across other age and background-diverse populations. Thus, further formative research using hypothetical vignettes or scenarios with concrete examples of HCS study design and procedures are recommended to ground participant attitudes in specific circumstances, thereby enhancing the study’s ability to predict actual enrollment, reduce interpretation variability and contextualize acceptance of HCS in Vietnam. Lastly, our study was conducted in a university setting, raising a concern that students might feel coerced to participate. To address this issue, we clearly assured students verbally and in writing that their participation was entirely voluntary, and that UMP staff had no access to the participant list or involvement in study administration. Our study activities were strictly supervised by UMP Ethics Committee. A separate publication describing the students’ experience participating in the SEED project is forthcoming, but informal feedback indicates coercion was not an issue.

## Conclusions

This study on the views of students training to be healthcare professionals, who might be involved in HCS as future clinicians, scientists, or even as participants, provides a grounded understanding to support HCS research planning with early and thoughtful consideration of participants’ expectations without compromising research ethics. Given the ethically sensitive nature of intentionally exposing healthy people to an infectious pathogen, HCS raise many complex and controversial questions surrounding benefit sharing, exploitation, participant selection and undue inducement of financial payment. Therefore, it is imperative that HCS should be conducted rigorously under ethics committee oversight, in compliance with national and international frameworks and regulations. Integrating social science research and fostering early and continuous engagement with the wider community are also essential to enhance the public acceptance of HCS, facilitate recruitment efforts, reduce early study withdrawals and consolidate participant commitment.

## Supporting information

S1 Appendix Comprehensive survey. (DOCX)

S2 Appendix Guidances for focus group discussion and in-depth interviews. (DOCX)

S3 Appendix Assessment of students’ acceptability of human challenge studies, clinical trials and observational studies. (DOCX)

## References

[1] Jamrozik E, Selgelid MJ. Human challenge studies in endemic settings: ethical and regulatory issues. Springer Cham; 2021. doi: 10.1007/978-3-030-41480-1

[2] Bodhidatta L, Pitisuttithum P, Chamnanchanant S, Chang KT, Islam D, Bussaratid V, et al. Establishment of a Shigella sonnei human challenge model in Thailand. Vaccine. 2012;30(49):7040–5. doi: 10.1016/j.vaccine.2012.09.061 23069701 PMC3732056

[3] Elliott AM, Roestenberg M, Wajja A, Opio C, Angumya F, Adriko M, et al. Ethical and scientific considerations on the establishment of a controlled human infection model for schistosomiasis in Uganda: report of a stakeholders’ meeting held in Entebbe, Uganda. AAS Open Res. 2018;1:2. doi: 10.12688/aasopenres.12841.2 30714021 PMC6358001

[4] Gibani MM, Jin C, Darton TC, Pollard AJ. Control of Invasive Salmonella Disease in Africa: Is There a Role for Human Challenge Models?. Clin Infect Dis. 2015;61(suppl_4):S266–71. doi: 10.1093/cid/civ673 PMC4596929 26449941

[5] Gordon SB, Rylance J, Luck A, Jambo K, Ferreira DM, Manda-Taylor L, et al. A framework for Controlled Human Infection Model (CHIM) studies in Malawi: Report of a Wellcome Trust workshop on CHIM in Low Income Countries held in Blantyre, Malawi. Wellcome Open Res. 2017;2:70. doi: 10.12688/wellcomeopenres.12256.1 29018841 PMC5627502

[6] Hodgson SH, Juma E, Salim A, Magiri C, Njenga D, Molyneux S, et al. Lessons learnt from the first controlled human malaria infection study conducted in Nairobi, Kenya. Malar J. 2015;14:182. doi: 10.1186/s12936-015-0671-x 25927522 PMC4416324

[7] Kaewkungwal J, Adams P, Sattabongkot J, Lie RK, Wendler D. Conducting human challenge studies in LMICs: A survey of researchers and ethics committee members in Thailand. PLoS ONE. 2019;14(10):e0223619-e. doi: 10.1371/journal.pone.0223619 PMC6786649 31600282

[8] Kestelyn E, Le Phuong C, Ilo Van Nuil J, Dong Thi HT, Minh Nguyen N, Dinh The T, et al. Expert voices and equal partnerships: establishing Controlled Human Infection Models (CHIMs) in Vietnam. Wellcome Open Res. 2019;4:143. doi: 10.12688/wellcomeopenres.15337.1 31681857 PMC6820821

[9] Vaz M, Timms O, Rose A, Manesh A, Bhan A. Consultation on the feasibility and ethics of specific, probable controlled human infection model study scenarios in india: a report. Indian J Med Ethics. 2019;4(3):238–42. doi: 10.20529/IJME.2019.030 31213422

[10] Pham-Tram V, Nguyen NM, Heatherley S, Vu Duy K, Vu BH, Pham Thi Huong G, et al. Exploring attitudes to research involving human subjects among Vietnamese university students: establishing a prospective longitudinal mixed-methods student cohort at the University of Medicine and Pharmacy at Ho Chi Minh City. Wellcome Open Res. 2024;8:473. doi: 10.12688/wellcomeopenres.19632.2 39114816 PMC11303948

[11] Le Phuong C, Pham-Tram V, Huynh Thuy Phuong H, Thi Hoai Tam D, Minh Nguyet N, Dinh The T, et al. Vietnamese university students’ perceptions and attitudes toward participation in clinical research: mixed methods study. J Particip Med. 2026;18:e86269. doi: 10.2196/86269 PMC12946779 41678793

[12] R Core Team. R: A language and environment for statistical computing. 2021.

[13] OpenAI. ChatGPT.

[14] Jae Jeong H. “The level of collapse we are allowed: comparison of different response scales in Safety Attitudes Questionnaire”. Biometr Biostat International J. 2016;4(4). doi: 10.15406/bbij.2016.04.00100

[15] Brant R. Assessing proportionality in the proportional odds model for ordinal logistic regression. Biometrics. 1990;46(4):1171–8. doi: 10.2307/2532457 2085632

[16] Benjamini Y, Hochberg Y. Controlling the false discovery rate: a practical and powerful approach to multiple testing. J R Stat Soc Series B: Stat Methodol. 1995;57(1):289–300. doi: 10.1111/j.2517-6161.1995.tb02031.x

[17] Pearson K. X. On the criterion that a given system of deviations from the probable in the case of a correlated system of variables is such that it can be reasonably supposed to have arisen from random sampling. London Edinburgh Dublin Philos Magaz J Sci. 1900;50(302):157–75. doi: 10.1080/14786440009463897

[18] Ranganathan P, Caduff C, Frampton CMA. Designing and validating a research questionnaire - Part 2. Perspect Clin Res. 2024;15(1):42–5. doi: 10.4103/picr.picr_318_23 38282630 PMC10810057

[19] Cohen P, Cohen J, Aiken LS, West SG. The problem of units and the circumstance for POMP. Multivariate Behav Res. 1999;34(3):315–46. doi: 10.1207/s15327906mbr3403_2

[20] Braun V, Clarke V. Using thematic analysis in psychology. Qual Res Psycholo. 2006;3(2):77–101. doi: 10.1191/1478088706qp063oa

[21] Naemiratch B, Kulpijit N, Ruangkajorn S, Day NPJ, Prachumsri J, Cheah PY. Experiences, perceptions and ethical considerations of the malaria infection study in Thailand. BMC Med Ethics. 2025;26(1):14. doi: 10.1186/s12910-024-01160-7 39875955 PMC11773921

[22] Jao I, Marsh V, Che Chi P, Kapulu M, Hamaluba M, Molyneux S, et al. Deliberately infecting healthy volunteers with malaria parasites: Perceptions and experiences of participants and other stakeholders in a Kenyan-based malaria infection study. Bioethics. 2020;34(8):819–32. doi: 10.1111/bioe.12781 32643809 PMC7689838

[23] Njue M, Njuguna P, Kapulu MC, Sanga G, Bejon P, Marsh V, et al. Ethical considerations in Controlled Human Malaria Infection studies in low resource settings: Experiences and perceptions of study participants in a malaria Challenge study in Kenya. Wellcome Open Res. 2018;3:39. doi: 10.12688/wellcomeopenres.14439.2 29806038 PMC5954342

[24] Dabira ED, Fehr A, Beloum N, Van Geertruyden J-P, Achan J, Erhart A, et al. Perceptions and acceptability of the controlled human malaria infection (CHMI) model in The Gambia: a qualitative study. Sci Rep. 2023;13(1):8708. doi: 10.1038/s41598-023-35752-x 37248260 PMC10226436

[25] Kraft SA, Duenas DM, Kublin JG, Shipman KJ, Murphy SC, Shah SK. Exploring ethical concerns about human challenge studies: a qualitative study of controlled human malaria infection study participants’ motivations and attitudes. J Empir Res Hum Res Ethics. 2019;14(1):49–60. doi: 10.1177/1556264618820219 30585505

[26] Oguti B, Gibani M, Darlow C, Waddington CS, Jin C, Plested E, et al. Factors influencing participation in controlled human infection models: a pooled analysis from six enteric fever studies. Wellcome Open Res. 2019;4:153. doi: 10.12688/wellcomeopenres.15469.1

[27] Kunda-Ngándu EM, Chirwa-Chobe M, Mwamba C, Chipungu J, Ng’andu E, Mwanyungwi Chinganya H, et al. Exploring willingness to participate in future Human Infection Studies in Lusaka, Zambia: A nested qualitative exploratory study. PLoS One. 2021;16(7):e0254278. doi: 10.1371/journal.pone.0254278 34242320 PMC8270142

[28] Mtunthama Toto N, Gooding K, Kapumba BM, Jambo K, Rylance J, Burr S, et al. “At first, I was very afraid”-a qualitative description of participants’ views and experiences in the first Human Infection Study in Malawi. Wellcome Open Res. 2021;6:89. doi: 10.12688/wellcomeopenres.16587.2 35187267 PMC8825950

[29] Browne JL, Rees CO, van Delden JJM, Agyepong I, Grobbee DE, Edwin A, et al. The willingness to participate in biomedical research involving human beings in low- and middle-income countries: a systematic review. Trop Med Int Health. 2019;24(3):264–79. doi: 10.1111/tmi.13195 30565381 PMC6850431

[30] Roestenberg M, Hoogerwerf M-A, Ferreira DM, Mordmüller B, Yazdanbakhsh M. Experimental infection of human volunteers. Lancet Infect Dis. 2018;18(10):e312–22. doi: 10.1016/S1473-3099(18)30177-4 29891332

[31] World Health Organization. WHO guidance on the ethical conduct of controlled human infection studies. Geneva: World Health Organization; 2021.

[32] Bambery B, Selgelid M, Weijer C, Savulescu J, Pollard AJ. Ethical criteria for human challenge studies in infectious diseases. Public Health Ethics. 2016;9(1):92–103. doi: 10.1093/phe/phv026 29731811 PMC5926904

[33] Miller FG, Grady C. The ethical challenge of infection-inducing challenge experiments. Clin Infect Dis. 2001;33(7):1028–33. doi: 10.1086/322664 11528576

[34] Blom JMC, Rivi V, Tascedda F, Pani L. Building trust in clinical research: a systems approach to ethical engagement and sustainable outcomes. Front Pharmacol. 2025;16:1570899. doi: 10.3389/fphar.2025.1570899 40741004 PMC12307415

[35] Abo Y-N, Jamrozik E, McCarthy JS, Roestenberg M, Steer AC, Osowicki J. Strategic and scientific contributions of human challenge trials for vaccine development: facts versus fantasy. Lancet Infect Dis. 2023;23(12):e533–46. doi: 10.1016/S1473-3099(23)00294-3 37573871

[36] Williams B, Morrison J, Wilkinson D, Savulesc J. The ethics of controlled human infection model studies for mitigating pandemic risks. 2023. https://www.uehiro.ox.ac.uk/article/report-the-ethics-of-controlled-human-infection-model-studies-for-mitigating-pandemic-risks

[37] Grimwade O, Savulescu J, Giubilini A, Oakley J, Osowicki J, Pollard AJ, et al. Payment in challenge studies: ethics, attitudes and a new payment for risk model. J Med Ethics. 2020;46(12):815–26. doi: 10.1136/medethics-2020-106438 32978306 PMC7719900

[38] Lynch HF, Darton TC, Levy J, McCormick F, Ogbogu U, Payne RO, et al. Promoting ethical payment in human infection challenge studies. Am J Bioeth. 2021;21(3):11–31. doi: 10.1080/15265161.2020.1854368 33541252

[39] Young B, Giubilini A, Sam XH, Lysaght T, Tambyah PA, Schaefer GO, et al. An Ethical analysis of public attitudes towards controlled human infection studies in singapore: acceptability and payment. Asian Bioeth Rev. 2025;18(1):117–34. doi: 10.1007/s41649-024-00335-z 41586173 PMC12824035

[40] Emanuel EJ, Grady C, Crouch RA, Lie RK, Miller FG, Wendler D. The Oxford Textbook of Clinical Research Ethics. Oxford University Press; 2008.

[41] Holzer F. Bridging exploitation and structural injustice: the example of clinical research. Revista Latinoamericana de Filosofía Política. 2020;9(1).

[42] Iltis AS, MacKay D. The Oxford Handbook of Research Ethics. Oxford University Press; 2024. 2020. doi: 10.1093/oxfordhb/9780190947750.001.0001

[43] London AJ. For the common good: philosophical foundations of research ethics. Online ed. Oxford University Press. 2022. doi: 10.1093/oso/9780197534830.001.0001

[44] Shah SK. Intentionally infecting humans: is it ethical?. Oxford: Oxford University Press. 2026. doi: 10.1093/9780197667927.001.0001

[45] Moreno JD. It’s not about the money. Am J Bioeth. 2001;1(2):46–7. doi: 10.1162/152651601300169059 11951888

[46] Dresser R. Payments to research participants: the importance of context. Am J Bioethics. 2001;1(2):47. 10.1162/152651601300169068 11951889

