# Supplementary material for: Balancing altruism, risk and ethics: Vietnamese students’ views on human challenge studies
Source: PLoS One. 2026 Sep 18;21(9):e0347941. doi: 10.1371/journal.pone.0347941 (PMC13588357; doi:10.1371/journal.pone.0347941)
Supplement: Supplementary file 2 [file pone.0347941.s002.docx]

**BALANCING ALTRUISM, RISK AND ETHICS: VIETNAMESE STUDENTS’ VIEWS ON HUMAN CHALLENGE STUDIES.**

**Supporting information:**

**S2 Appendix - Guidance for focus group discussion and in-depth interviews.**

**Aim:**

To explore students’ perspectives and attitude toward human challenge studies (HCS) in Vietnam acceptability)

***Specific objective:***

The purpose of this series of focus group discussions (FGDs) and in-depth interviews (IDIs) is to explore the decision-making of students as the lens through which to interpret their perception towards HCS. The specific objectives are to:

- elicit and analyze the perceptions of students on HCS;
- explore the ways in which students will make decision to participate HCS;
- gain insight into the positioning of balance between benefits and burdens related to HCS;
- develop a contextual understanding of relationship between participants’ values and their participation into HCS;
- Explore students’ motivation to join into HCS

# I. FOCUS GROUP DISCUSSIONS GUIDE

## Topic 1: Introduction of general human challenge models

*Aim: To explore students’ perception of the concept of* *the human challenge study*

1. What do you personally think of the idea of infecting people with pathogens in research?

- How do you feel about this concept? Could you please explain more about your feelings?

2. What might be the benefits/value of intentionally infecting people with pathogens?

3. What concerns or worries do you have about intentionally infecting people with pathogens?

*Probe:*

- Which of these risks and burdens concern you the most? Which concern you the least?

4. How do you think the community/public might feel/think of the idea of infecting people with pathogens?

*Probe*: Asking about social and ethical acceptability

## Topic 2: Motivation to join human challenge models

*Aim: To explore students’ motivation to join HCS and related factors*

1. What is your first thought if you are invited to participate in HCMs? Would you participate in this type of study?

- If the answer is no, please tell me why you choose not to participate?
- Fear? Worries? Concerns?
- If the answer is yes:
- What is your motivation to participate in such a study?

2. What factors do you need to think about before deciding to participate?

- What’s the order of those factors? What do you consider first, what can come later?

3. To what extent are you willing to participate in the HCS?

- In your opinion, where is the limit of research that you can accept? This means that if the research entails something (factors) over that limit, you will totally not consider participate.
- What aspect in HCS that you may find it acceptable to you? (in term of health, care, reimbursement, benefits, risks, etc.?
- If you decide to participate in HCS, do you think your family could benefit from your participation in HCS?

4. What do you think is the most important factor that would motivate young and healthy people to participate in HCS?

*Probe:*

- What importance/value do you think young and healthy people place in their motivation to participate in the HCS?

## Topic 3: Perception of HCS benefits and value

*Aim: To explore students’ perception of HCS benefits and value*

1. What might be the benefits of HCS?

*Probe:*

- Benefits to participants personally
- Benefits to community and society
- Which subjects are the most beneficent from the results of HCS?

2. What do you think about the value participants place in their involvement in HCS?

3. What do think the value of HCS in comparison to other types of clinical research?

*Probe*: In terms of

- Knowledge generation
- Enhancing community’s health benefits
- Control of the infectious diseases of interest (in Vietnam or globally)
- Capacity and technology development
- Medical research and practice

4. Are there any economic benefits if we conduct HCS?

- If the answer is No, ask the reason why?
- If the answer is Yes, what are potential economic benefits? Who will be the beneficiaries of these economic/financial benefits from HCS?

5. Is the implementation of HCS appropriate with the medical development level of Vietnam? Why do you think so?

## Topic 4: Perception of HCS risk

*Aim: To explore students’ perception of risk related HCS*

1. What are your worries about/concerns the risk of HCS?

2. What are the impacts on health that you may foresee / are aware of that HCS entails?

Probe:

- Where do these health impacts come from?
- How does the risk of HCS compare to other types of clinical research, such as observational studies or clinical trials?

3. What are the concerns from the community regarding HCS?

- Probe for the risk on community and environments

4. What could be done to mitigate those concerns?

Probe:

- What is the minimum level of protection that the investigators should provide to the participants?

## Topic 5: Financial considerations in HCS

*Aim: To explore students’ attitude toward financial considerations in HCS*

1. What factors should be accounted for to remunerate for participation?

*Probe:*

- What would participants be reimbursed and compensated for if they participate in HCS?
- How much do you feel is suitable compensation for participation in a human challenge trial?

2. What issues/values (related to ethics/fairness/human rights…) should be concerned when estimating compensate participants?

3. If we offered compensation for participating in this type of research, how would it influence your decision?

4. To what extent is getting incentives to participate in a human challenge study important to you?

## Topic 6: Involvement of family members and others in HCS participation decision-making

*Aim: To explore the influences of family members and public attitude toward HCS participation.*

1. What are the community’s attitude and opinions about HCS?

2. Who would you ask for advice before deciding to participate in HCS? How do you think they can react?

3. To what extent do your family/parents’ opinions influence what you consider participating in HCS?

- What happens if you don’t follow your parents/those people’s advice?

4. If you agree to join the human challenge models, would you tell others about your participation?

- Who would you choose to tell this to? (Family members? Friends? Neighbors?)
- How do you think they can react?

5. How would participating in the HCS clash with your cultural practices and beliefs around health?

- Are there anyone in your community against participating in human challenge models? Who are they? What reasons would be?

# II. IN-DEPTH INTERVIEWS GUIDE

|  | **Interviewing Guidance** |
| --- | --- |
|  | **Warm up** |
| **Students’ perceptions of the idea of infecting people with pathogens in research** | 1. What do you think of the HCT concept? Or about infecting people with pathogens in research?  Probe:   - How do you feel about this concept? - Could you please explain where your feelings come from? - Have you ever changed your feelings/thoughts during your participation in the SEED project? Why and How? |
| **Motivation to participate in HCS** | 2. If you are invited to participate in a HCS, would you be willing to participate in this study?   - If the answer is no: Please tell me why you choose not to participate. - Are there any worries/concerns/reasons that may make you hesitant or stop you from joining a human challenge trial? Please explain. - If the answer is yes: What is your motivation to participate in such a study? |
| **The ways in which students will make decisions to participate in HCS including some factors contributes to decision making.** | 3. What contributes to your decision-making of participation into HCS?  *Probe:*   - What’s the order of those factors? What do you consider first? What can come later? - You have said that you would/would not participate in HCS. Are there any circumstances you would change your mind? What are they?   *(Note: If the interviewee mentions the benefits/risk /burden, the interviewer will probe the questions below)* |
| **Perceptions about benefits and social value of HCS** | 4. What might be the benefits/value of HCS?   - Benefits to participants personally - Benefits to community and society   5. What importance/value do you think participants place in their involvement in HCS?   - To what extent do these benefits/values influence your motivation to participate in HCS? |
| **Perceptions about HCS risk to participants** | 6. To your understanding, what are your worries or concerns about the risk to HCS participants?  *Probe:* Compared to other clinical research types:   - How does the risk of HCS compare to other types of clinical research, such as observational studies or clinical trials? - Is your willingness to participate in HCS different? Why do you think so? |
| ***Health consequence risk*** | 7. What are the impacts on health that you may foresee / are aware of that one HCT entails?  *Probe:*   - Where do these health impacts come from?   *[If the students mentioned ‘symptoms’ or complications]*   - What symptoms or complications of infection you may find tolerable/acceptable? and which not? - Probe: type, duration, magnitude; like short-term, long-term effects: which temporal risk might you concern/weigh more?   8. To what extent are the severity of the possible health impacts acceptable to you to participate in HCS?   - Please tell me the minimum and maximum levels you think you can accept. - What are the worst scenarios / health impacts that you think it happens to one joining the trial? In which situation may you accept? - Probe into risks of irreversible, incurable, or possibly fatal infections   9. How likely do you think you will experience these health impacts if you participate in HCS? To what extent is the likelihood of these impacts acceptable to you to participate in HCS? |
| ***Disease-related risk*** | 10. What are the characteristics of diseases that may affect your decision to join? Do you think the risks of HCS will vary among different types of diseases?  *Probe into:*   - *Disease prevalence:* - Outside of the study, do you think the risk that you would be infected with the disease within 6 months will influence your decision to participate in HCS? - *Level of understanding* - Do the levels of understanding about the disease influences your decision to participate in HCS - To what extent does the specific ‘rescue therapy’ to those pathogens affect your decision to join? - What do you think about the diseases that already have specific treatment (therapeutic)? - What about diseases that haven’t had any specific treatments (therapeutic)? - Are there any chance those two types of treatment (specific vs. non-specific) affect your decision differently? - *Transmission route* - How do the transmission routes influence your willingness to participate in HCS? - Do you think the risk of HCS will vary if the investigators use different transmission routes? - *Type of pathogens* - Do you think the risk of HCS will vary among different types of micro-organism? - To what extent is the levels of attenuation/modification of pathogen (attenuated vs wild type) that you may find acceptable? |
| ***Risk of investigational vaccines/drugs*** | 11. What do you think about the risk related to investigational new drugs/vaccines in HCS?  *Probe:* Compared to risk of clinical trials? |
| **Perception on Burden/inconveniences** | 12. How do you think taking part in HCS could affect your life today or in the future?  *Probe:*   - What aspects of your life will be most affected by your participation in the HCS, and how?   13. What are the burdens/inconveniences you might experience during the participation in HCS?   - Which of these burdens concern you the most? Which ones concern you the least?   14. How would you feel about being in strict isolation in a specialized isolation unit?   - What is the maximum length of time you can be isolated? - What would be the potential problems if you have to be isolated in a medical unit?   15. To what extent are the levels of burdens/inconveniences of the potential impacts acceptable to you? How do the levels of burdens/inconveniences influence your desire to participate in HCS? |
| **Third-party risk** | 16. What other risks that you may have due to your participation that can affect environment or community? |
| **Safety considerations** | 17. What factors should be considered to ensure HCS are conducted safely?  18. If you agree to participate in HCS, what is the minimum level of protection that you expect the investigators to provide to the participants?  *Probe:*   - What is your opinion about the medical care that participants should receive during and after HCS? - How much care do you expect from the researchers if you agree to participate in the HCS? |
| **Students’ expectations to investigators** | 19. Once you agree to participate in the human challenge trial, you might need to be confined for a period of time. What conditions would the study need to meet to ensure that you’re comfortable when you’re enrolled in the study? |
| **Financial considerations** | 20. To what extent do the financial benefits influence your motivation to participate in the HCS?  *Probe:*   - To what extent is getting incentives to participate in a human challenge trial important to you?   21. What factors should be accounted for to remunerate for participation?  *Probe:*   - What would you need to be compensated for if you participated in the HCS? - How much do you feel is suitable compensation for participation in a human challenge trial? |
| **Trust to research team** | 22. If you decide to take part in research, who or what do you place your trust in? Why?  23. What make you trust (or not) the investigators or institution?  *Probe:*   - What elements do you consider? - In which situation could they make you feel untrustworthy? |
| **Involvement of family members/significant others** | 24. If you are invited to participate in HCS, who make decisions about what you should or should not do?  25. Who would you ask for advice before deciding to participate in HCS? How do you think they can react?  26. To what extent do advice from your parents/ family members/significant others influence your desire to participate in HCS?   - What happens if you don’t follow your parents/those people’s advice?   27. How do you think you will be treated by your family and friends if they know that you have been participate in HCS? |
| **Attitude toward information from internet and media** | 28. If you are invited to HCS, will you search for information from the internet or media?  *Probe:*   - What information do you want to know? Where will you search for information? - To what extent do the information from the internet influence your desire to participate in HCS? - Would you change your decision if the information from the internet differs from other sources (i.e., family or friends’ advice)? Why? |
| **View on participate in Dengue HCS** | 28. How do you feel about being deliberately infected with the Dengue virus?  *Probe:*   - How do you think you will be treated by your family and friends if they know that you have been infected with the Dengue virus? |
| **COVID-19 pandemic** | 29. Do your feelings/thought toward HCS change after COVID-19 pandemic? Why? |
| **Ending** | 30. Considering all factors related to participation to HCS, which one takes your further attention? Why?   - What is the order of those factors influencing your desire to participate in HCS? - What do you consider first? What can come later?   31. What else would you need to consider before deciding to volunteer to taking part? |
|  | Thank you for participating in this interview |
