# Supplementary material for: Balancing altruism, risk and ethics: Vietnamese students’ views on human challenge studies
Source: PLoS One. 2026 Sep 18;21(9):e0347941. doi: 10.1371/journal.pone.0347941 (PMC13588357; doi:10.1371/journal.pone.0347941)
Supplement: Supplementary file 3 [file pone.0347941.s003.docx]

# BALANCING ALTRUISM, RISK AND ETHICS: VIETNAMESE STUDENTS’ VIEWS ON HUMAN CHALLENGE STUDIES.

# Supporting information:

# S3 Appendix - Assessment of students’ acceptability of human challenge studies, clinical trials and observational studies.

We calculated an overall score of students’ acceptability of human challenge studies (HCS), clinical trials and observational studies, by using seven Likert scale questions of comprehensive survey: (1) “It is important for society that this type of research is carried out.”; (2) “It is safe to participate in this type of research.”; (3) “I would be happy to participate in this type of research myself.”; (4) “For myself, currently, it would be important to get permission from my parents in order for me to participate in this type of research”; (5) “Researchers must check that participants really understand the details of the research (e.g., with a short test) before they enroll them’; (6) “Financial benefits should cover all costs, but no added incentives should be given.”; and (7) “Additional incentives should be given.”. The Likert criteria of strongly disagree to strongly agree were converted to numerical representation (1-5). Prior to inclusion in the composite score, the questionnaire was tested for reliability using the Cronbach’s alpha. In addition, this was also reviewed by experts to ensure content validity [1]. The question (4) was excluded because it reduced the internal consistency of the scale. Conceptually, this item was also less relevant to the study population, since all participants were adults and therefore did not require parental permission to participate in such research. Additionally, the question (6) was also excluded due to its overlap with question (7). Finally, five questions (1), (2), (3), (5), and (7) were included. The mean score across all 5 questions was calculated and the percentage of maximum possible (POMP) score was calculated, per study type at a group level, using the formula: POMP = ((observed score – minimum possible) / (maximum possible – minimum possible)) X 100 [2]. We used Wilcoxon signed-rank and Kruskal-Wallis tests with continuity correction to examine differences in POMP score across study type pairs (HCS versus Observational studies and HCS versus clinical trials; Figure 3B), and student groups stratified by sex, faculty, academic year, ethnicity or social-economic status (Table S1).

**Table S1: Students’ acceptability score of observational studies, clinical trials and human challenge studies.**

|  | **Observational studies** | | **Clinical trials** | | **Human challenge studies** | |
| --- | --- | --- | --- | --- | --- | --- |
|  | **Mean (SD)** | **p-value** | **Mean (SD)** | **p-value** | **Mean (SD)** | **p-value** |
| **Sex^1^** | | | | | | |
| Female | 77 (12) | 0.29 | 71 (11) | 0.11 | 69 (12) | 0.083 |
| Male | 78 (11) |  | 73 (11) |  | 71 (13) |  |
| **Faculty^1^** | | | | | | |
| General Medicine | 78 (11) | 0.31 | 73 (11) | 0.092 | 70 (12) | 0.81 |
| Public Health | 76 (13) |  | 70 (11) |  | 70 (12) |  |
| **Academic year at enrolment^1^** | | | | | | |
| First year | 76 (12) | 0.62 | 71 (11) | 0.064 | 69 (13) | 0.14 |
| Third year | 78 (12) |  | 73 (11) |  | 71 (12) |  |
| **Ethnicity^1^ *** | | | | | | |
| Kinh | 77 (12) | 0.19 | 72 (11) | 0.28 | 70 (12) | 0.37 |
| Hoa | 74 (5) |  | 74 (8) |  | 73 (9) |  |
| Others | 72 (13) |  | 67 (16) |  | 67 (16) |  |
| **Socio-economic status (self-reported)^2^ *** | | | | | | |
| Poor | 75 (15) | 0.56 | 72 (11) | 0.63 | 72 (7) | 0.71 |
| Average | 77 (11) |  | 72 (11) |  | 70 (12) |  |
| Wealthy | 70 (0) |  | 65 (7) |  | 63 (18) |  |
| Prefer not to answer | 78 (15) |  | 71 (11) |  | 72 (14) |  |
| *1: p-value from Wilcoxon signed-rank test.*  *2: p-value from Kruskal-Wallis test.*  **: Missing data: for ethnicity = 1; and for socio-economic status = 7.* | | | | | | |

**References:**

1. *Ranganathan P, Caduff C, Frampton CMA. Designing and validating a research questionnaire - Part 2. Perspectives in Clinical Research. 2024;15(1):42–45. doi:10.4103/picr.picr_318_23*
2. *Cohen P, Cohen J, Aiken LS, West SG. The Problem of Units and the Circumstance for POMP. Multivariate Behavioral Research. 1999/07/01 1999;34(3):315–346. doi:10.1207/S15327906MBR3403_2*
